# Reliability, validity, and clinical feasibility of a rapid and objective assessment of post-stroke deficits in hand proprioception

**DOI:** 10.1186/s12984-018-0387-6

**Published:** 2018-06-07

**Authors:** Mike D. Rinderknecht, Olivier Lambercy, Vanessa Raible, Imke Büsching, Aida Sehle, Joachim Liepert, Roger Gassert

**Affiliations:** 1 Rehabilitation Engineering Laboratory, Department of Health Sciences and Technology, ETH Zurich, Zurich, Switzerland; 2 Department of Neurorehabilitation, Kliniken Schmieder, Allensbach, Germany; 3 Lurija Institut, Konstanz, Germany

**Keywords:** Difference threshold, MCP, Metacarpophalangeal joint, Parameter Estimation by Sequential Testing, Psychophysics, Quantitative measurements, Robot-assisted assessment, Somatosensory function

## Abstract

**Background:**

Proprioceptive function can be affected after neurological injuries such as stroke. Severe and persistent proprioceptive impairments may be associated with a poor functional recovery after stroke. To better understand their role in the recovery process, and to improve diagnostics, prognostics, and the design of therapeutic interventions, it is essential to quantify proprioceptive deficits accurately and sensitively. However, current clinical assessments lack sensitivity due to ordinal scales and suffer from poor reliability and ceiling effects. Robotic technology offers new possibilities to address some of these limitations. Nevertheless, it is important to investigate the psychometric and clinimetric properties of technology-assisted assessments.

**Methods:**

We present an automated robot-assisted assessment of proprioception at the level of the metacarpophalangeal joint, and evaluate its reliability, validity, and clinical feasibility in a study with 23 participants with stroke and an age-matched group of 29 neurologically intact controls. The assessment uses a two-alternative forced choice paradigm and an adaptive sampling procedure to identify objectively the difference threshold of angular joint position.

**Results:**

Results revealed a good reliability (ICC(2,1) = 0.73) for assessing proprioception of the impaired hand of participants with stroke. Assessments showed similar task execution characteristics (e.g., number of trials and duration per trial) between participants with stroke and controls and a short administration time of approximately 12 min. A difference in proprioceptive function could be found between participants with a right hemisphere stroke and control subjects (*p*<0.001). Furthermore, we observed larger proprioceptive deficits in participants with a right hemisphere stroke compared to a left hemisphere stroke (*p*=0.028), despite the exclusion of participants with neglect. No meaningful correlation could be established with clinical scales for different modalities of somatosensation. We hypothesize that this is due to their low resolution and ceiling effects.

**Conclusions:**

This study has demonstrated the assessment’s applicability in the impaired population and promising integration into clinical routine. In conclusion, the proposed assessment has the potential to become a powerful tool to investigate proprioceptive deficits in longitudinal studies as well as to inform and adjust sensorimotor rehabilitation to the patient’s deficits.

## Background

Proprioception is of great importance for the control of fine and coordinated movements of the upper limb [1–4], and thus for activities of daily living [5–9]. Neurological injuries can affect proprioceptive function, and despite highly variable prevalence reported in the literature, it is estimated that about half of stroke patients suffer from impaired proprioception [10–15]. As there is growing evidence that somatosensory impairment leads to a poor prognosis for post-stroke functional recovery in patients with severe and persistent somatosensory dysfunction [16–19], proprioception in stroke patients has been receiving increased attention. Furthermore, proprioception has been shown to be a relevant predictor for the level of independence patients achieve at discharge [20, 21].

In order to investigate and better understand the role of proprioception in the recovery of neurological patients, for diagnosis as well as the design of therapeutic approaches, accurate and sensitive assessments are essential. Only very few assessments for proprioception are clinically used (e.g., up-down test [22, 23], dual joint position test [24], positional mimicry and finger finding [22, 25]), as, in contrast to other approaches, they are quick and easy to administer in a clinical setting. Unfortunately, these assessments are known to be highly subjective, use dichotomous or ordinal scales, and suffer from large variability due to the lack of standardized protocols and manual administration. This results in low inter-rater reliability, as well as ceiling effects [22, 26, 27]. Thus, they may be suitable for screening patients but not for assessing functional improvements [28]. According to the results of a cross-sectional survey, more than 50% of a sample of 172 occupational and physiotherapists agreed that current methods to assess somatosensory deficits should be improved [29], and assessments with finer degrees of movement (i.e., better controlled movements and finer grading) are required [30].

More quantitative approaches have been proposed, for example using simple apparatuses still requiring manual intervention of the examiner (e.g., using protractor scales [31–34] or objects to discriminate by grasping [35]). Furthermore, a large number of robotic approaches taking advantage of today’s actuation, control and sensing technology for better stimulus control [36] and using different assessment paradigms have been developed (e.g., matching and movement reproduction methods [14, 37–50], detection of passive motion [51–53] or perturbations [54–56], as well as difference threshold assessments [57–59]). An essential step when developing a new assessment is the evaluation of its psychometric and clinimetric properties (e.g., reliability, validity, sensitivity, feasibility, and clinical utility) and its potential confounds. To increase clinical acceptance, set-up and testing time, as well as the complexity and cost of the robotic devices should be reduced as much as possible. While some of these properties have been evaluated for matching and perturbation assessments, mostly targeting proximal joints such as shoulder and elbow (e.g., [38, 54, 60]), assessments estimating proprioceptive difference thresholds at finger joints have not been sufficiently optimized and evaluated so far.

The aim of our work is to investigate the reliability, feasibility, and validity of an existing robot-assisted assessment providing a quantitative outcome measure of the metacarpophalangeal (MCP) joint proprioception on a ratio scale [58] in participants with stroke and in an age-matched group of neurologically intact controls (NIC). The assessment is based on an objective two-interval two-alternative forced choice (2AFC) paradigm [61] and Parameter Estimation by Sequential Testing (PEST) [62], an adaptive sampling procedure used to determine perception thresholds. We examine the test-retest reliability for the impaired and unimpaired hands in participants with stroke, and evaluate the clinical feasibility and usability by investigating different task execution properties, such as duration, number of trials by comparing participants with stroke to NIC subjects, and confounds such as memory and inattention. We acknowledge that the terms inattention and neglect are often used interchangeably in the clinical realm. In the present manuscript we defer to how the term “inattention” is used in psychophysical testing and where it represents a time period of distraction from the task. Further, we use the term neglect to represent the common condition that occurs post-stroke and is characterized by a reduced sensory awareness of the side of the body and environment contralateral to the lesion. As a subanalysis, the differences between perception thresholds of participants with left hemisphere stroke (LHS), participants with right hemisphere stroke (RHS), and NIC subjects, and between both hands within participants with stroke (ipsilesional versus contralesional) and NIC subjects (dominant versus non-dominant) are compared (construct validity). Based on findings from other studies [14, 47, 63], we hypothesize that participants with stroke will have decreased proprioceptive performance compared to NIC subjects, and that the proportion of more severely affected participants with stroke may be larger in the RHS group than in the LHS group. Such findings could be related to existing evidence for a non-preferred arm advantage for proprioceptive feedback processing in neurologically intact subjects [40, 42, 64–67]. Furthermore, the robotic outcome measure is correlated to different clinical scales for somatosensation (concurrent validity). This study further discusses the potential of this assessment of proprioceptive deficits in participants with stroke for successful integration and use in a clinical setting.

**Fig. 1:**
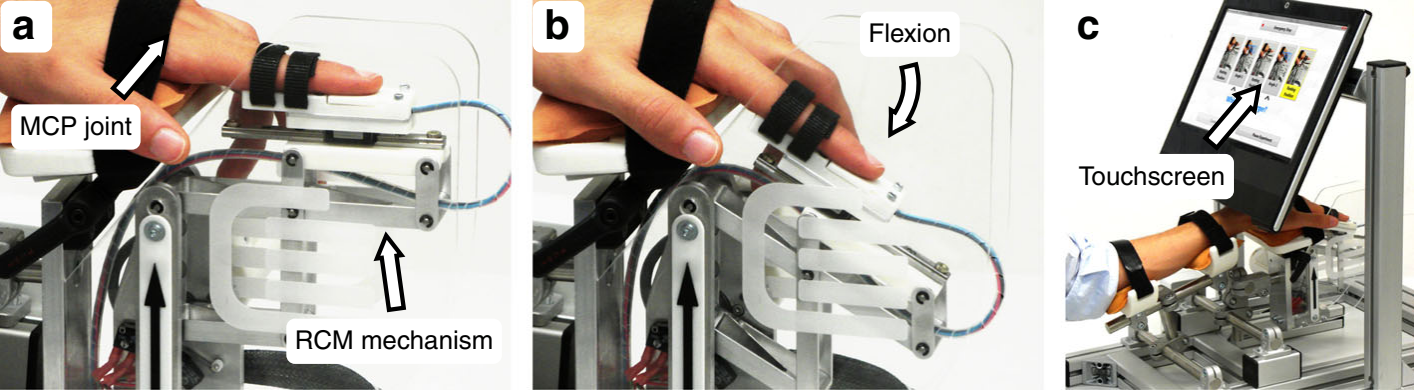
Robotic Sensory Trainer. **a** Rest position of the index finger. Side view on the remote-center-of-motion (RCM) mechanism of the apparatus used to apply passive movements around the metacarpophalangeal (MCP) joint. **b** Flexed position of the index finger. **c** Experimental setup with a touchscreen, covering the tested hand, for instructions and post-trial subject feedback on perceived stimuli

## Methods

### Subjects

Twenty-three participants with stroke were recruited and enrolled in this study. They had to be >2 weeks after their first clinical stroke (without an upper limit on post-stroke weeks). Participants with stroke were recruited on a patient-by-patient basis among the patients receiving an inpatient neurological rehabilitation at the Kliniken Schmieder Allensbach, Germany. Exclusion criteria for the participants with stroke were inability to detect any manually applied large passive finger movements. Other exclusion criteria regarding somatosensory deficits were not defined in this exploratory study to have a heterogeneous sample population with potentially a wide range of levels of proprioceptive deficits. Additional exclusion criteria were severe hand edema, high muscle tone—particularly in the flexor digitorum superficialis muscle and the flexor digitorum profundus muscle—evaluated with the Modified Ashworth Scale [68], or pain preventing the use of the robotic assessment tool, severe cognitive impairment, aphasia, and neglect. If participants with stroke had difficulties with understanding the goal of the study and the instructions, the Montreal Cognitive Assessment (MoCA) [69] was performed as a screening cognitive exam. In case of a value below 26 points on the MoCA scale, the participant was excluded. The presence of neglect symptoms was assessed by clinical observation. In case neglect was suspected, the Bells Test [70] was performed. Twenty-nine neurologically intact control (NIC) subjects within the same age range served as a control group. Only self-reported right handed subjects were included to avoid a handedness confound when comparing proprioceptive performance between the dominant and non-dominant hands in participants with LHS and RHS. Handedness was assessed with the Edinburgh Handedness Inventory (left handed: score <−40, right handed: score >40, ambidextrous otherwise) [71]. Participants with stroke were asked to evaluate their post-stroke handedness retrospectively. The study was approved by the institutional ethics committee of the University of Konstanz. All subjects gave signed, written informed consent in accordance with the Declaration of Helsinki before participating in the experiment.

### Robotic assessment of proprioception

#### Apparatus

The improved version of the Robotic Sensory Trainer was used in this study [58] (Fig. 1) to assess the proprioceptive difference threshold or limen (DL) in joint angle position of the MCP joint of the index finger. This robotic tool can provide well-controlled, passive MCP joint angle displacements in flexion and extension (Fig. 1a and b). The finger is inserted and attached to a sliding finger carriage mounted on a remote-center-of-motion mechanism allowing for a biomechanically correct movement around the MCP joint when the joint location is aligned with the prolongation of the black arrow on the device (Fig. 1a). The hand and forearm of the subject can be strapped to an adjustable support structure using Velcro ^*Ⓡ*^ bands, in an attempt to maximize comfort in any pathological hand and arm posture required by the tested subject. The flexion and extension displacements are controlled by software (LabVIEW, National Instruments, Austin, TX, USA) running on an all-in-one touchscreen computer covering the tested hand (Fig. 1c). The program not only runs the psychophysical protocol but also provides a subject interface on which the feedback (i.e., subject’s responses after each trial) can be provided.

#### Adaptive psychophysical procedure

A two-interval, 2AFC paradigm [61] in combination with the logarithmic version of the adaptive stimulus placement method PEST [62] were used as proposed and previously tested in a pilot study [58]. 2AFC should lead to more objective, sensitive and almost bias-free threshold estimates [61, 72]. PEST was selected among different stimulus placement methods due to its fast adaptation over a wide range of stimuli values resulting in an efficient assessment, which converges rapidly towards the desired threshold [58].

Each trial consisted of two consecutive passive index finger movements to different MCP joint flexion angles applied by the robotic apparatus. Each movement sequence (i.e., interval) started at a horizontal finger position (referred to as rest position) as depicted in Fig. 1a. Each flexion movement lasted 1 s and the finger was kept at the MCP flexion angle for 1.5 s before moving back to resting position. Each movement followed a natural minimum jerk trajectory [73]. After the two intervals, the subject was asked to indicate on the touchscreen (Fig. 1c) which of the two angular movements was perceived as larger (2AFC paradigm).

The angular difference (referred to as stimulus level *x*) between the two presented angles of one trial was always defined as positive and determined by the PEST algorithm [62] taking past responses into account. The two angles were symmetrically arranged around a reference angle of 20° MCP flexion and presented in randomized order. The range of *x* was limited to flexion movements and, by the mechanical constraints of the device, to [0°, 40°]. As starting parameters of the PEST algorithm, a start level *x*_0_ and a start step of 5.5° and 2°, respectively, were chosen. The other PEST parameters *W*=1 and *P*_*t*_=0.75 were chosen for the Wald sequential likelihood-ratio test [74] leading to a convergence towards 75% correct responses. This set of parameters was successfully tested in a previous pilot study with young NIC subjects [58]. The robotic assessment was terminated as soon as one of the three termination conditions were fulfilled: (i) minimum step of ±0.1°, (ii) 20 consecutive trials at same level, or (iii) a maximum number of trials defined in the experimental protocol reached.

#### Primary outcome measure: difference limen estimation

In theory, PEST should directly provide the difference limen (DL, in the literature sometimes also referred to as difference threshold or just-noticeable difference) by convergence. According to the original concept, the level (at which no trials are actually run) called for by the last small step can be used as the threshold estimate [62]. However, depending on the choice of termination criteria and parameters, it can occur that PEST does not converge within the given number of maximum trials. Furthermore, it is possible that periods of lack of attention towards the end of the assessment may lead to partial divergence from the threshold, resulting in poorer estimates. These issues can be partly addressed by using hybrid procedures: determining the test levels with the PEST algorithm and estimating the final threshold estimate from a parametrized psychometric function fitted on the data of the entire PEST sequence [75]. It could be shown in our previous work that the DL at the MCP joint estimated by this fitting method strongly correlated with the converged values provided by PEST [58] with the advantage of being more robust and providing also reasonable estimates in the above mentioned cases.

Loss of attention during psychophysical assessments, in particular in those using two-interval 2AFC, can be a confound leading to considerable bias, due to altered perception, especially as attention deficits are likely to be present in the stroke population [76, 77]. To address this issue, a method was developed allowing to identify the onset and end of sustained inattention periods in PEST sequences, to exclude this interval of biased data before fitting the psychometric function and calculating the outcome measure [78]. This method can significantly reduce estimation errors by up to around 75%, even in sequences of less than 100 trials, as demonstrated in computer simulations and tested on behavioral data [78]. Thus, before estimating the DL in the present study, this method was applied *post-hoc* to each recorded PEST sequence.

In the present work, the following sigmoidal psychometric function *ψ*(*x*) was fitted to the proportion of correct responses at stimulus levels *x* using a Maximum Likelihood criterion [79]:

1 $$ \psi\left(x;\alpha,\beta,\gamma,\lambda\right) = \gamma + \left(1-\gamma-\lambda\right) F\left(x;\alpha,\beta\right) ~\text{.}  $$

In this work, the generic sigmoid function *F*(*x*;*α*,*β*) of the equation above corresponded to a cumulative Gaussian function *F*_*Gauss*_(*x*;*μ*,*σ*). The parameter *μ* corresponds to the inflection point of *ψ*(*x*) and *σ* is inversely proportional to the slope at the inflection point. According to the 2AFC paradigm, the guessing rate *γ* was set to 0.5. The lapse rate *λ* (taking into account stimulus-independent errors also referred to as lapses) was allowed to vary within [ 0,0.1], in order to reduce estimation bias [80]. Since the inflection point *μ* depends on the lapse rate *λ*, the DL was defined as outcome measure at *x*_*T*_=*ψ*^−1^(0.75).

### Clinical assessments

Proprioceptive function was assessed based on the up-down test described by [22]. The distal phalanx of the index finger was moved up or down, 5 times each, in random order. Participants with stroke reported the direction of the movement verbally in absence of vision of the tested finger. The final score (0–10) consisted of the number or correctly identified movement directions.

In addition, other somatosensory modalities were tested. Topesthesia (localization of touch) was tested by manually stroking the dorsal side of the fingers (2x per finger, random order). The outcome measure was the number of correctly identified fingers (0–10). Von Frey hairs (OptiHair_2_, MARSTOCKnervtest, Schriesheim, Germany) were used to assess the absolute tactile perception threshold on the fingertip of the index finger. The score was computed by taking the geometric mean of the reverse values (5 suprathreshold and 5 subthreshold) of the descending staircase according to [81], on a scale from 1 (0.25 mN) to 12 (512 mN). Pallesthesia (sensation of mechanical vibration) was assessed using a 64 Hz, graduated Rydel-Seiffer tuning fork (Martin, Tuttlingen, Germany) [82] on the MCP joint of the index finger. The sensibility was scored from 0–8 in steps of one with 0 corresponding to no sensation at all. Stereognosis (ability to recognize objects by using only tactile information) was assessed with the subscale of the Nottingham Sensory Assessment [22]. The outcome was the number of correctly identified objects (0–10).

Attention and working memory were assessed using the backward recitation condition of the Digit Span subtest of the Wechsler Adult Intelligence Scale ^*Ⓡ*^ - Third Edition (WAIS ^*Ⓡ*^-III) [83], where participants with stroke were asked to recite an auditorily presented series of digits backwards. Two trials with random numbers were consecutively performed for each digit span (2–6 numbers, in increasing order). The total score consisted of the total number of correctly recited digit spans ranging from 0–12.

### Experimental protocol

Both, participants with stroke and NIC subjects performed the robotic proprioception assessment of both hands (randomized order) in one session. For the participants with stroke, the maximum number of trials for the PEST algorithm was set to 60 trials, which should correspond to an assessment duration of around 15 min according to a pilot study in young NIC subjects [58]. This number of trials was selected to allow a future integration of the assessment into clinical routine, and because longer assessments could be too strenuous for participants with stroke due to the cognitive demand (e.g. attention to the task). As these points were less critical for NIC subjects, the maximum number of PEST trials was set to 120 trials. This allowed evaluating the appropriateness of the chosen parameters of the PEST procedure. To investigate test-retest reliability, the robotic test was conducted in participants with stroke in a second session not more than 4 days after the first session. For a subset of 10 of the 23 recruited participants with stroke, clinical assessments (of both hands where applicable) were performed in a separate session by a different therapist. The therapists were blinded to the outcomes of the other assessments. No clinical assessments were conducted in NIC subjects due to ceiling effects. All assessments took place at the Kliniken Schmieder Allensbach, Germany.

### Data analysis

To evaluate the test-retest reliability (in participants with stroke), the intraclass correlation coefficient *ICC*(2,1) (two-way layout with random effects for absolute agreement) [84], as well as its 95% confidence interval (CI), standard error of measurement *SEM*, and smallest real difference *SRD* (sometimes referred to as minimal detectable change *MDC*) were computed according to [85] and [86]. Systematic bias was analyzed by calculating the mean difference $\bar {d}$ between the two test occasions and its 95% CI, and by visualization in a Bland-Altman plot [85]. The reliability analysis was performed separately for the impaired and unimpaired hand.

To compare the outcome measures of NIC subjects to participants with stroke and to create models of neurologically intact performance, the PEST sequences of the NIC subjects were truncated to 60 trials to be of same length as for the participants with stroke. Furthermore, outliers in the NIC group were identified according to Tukey’s rule and excluded from all statistical analysis.

Outcome measures from the robotic assessment were compared between the left and right hand of NIC subjects (paired test), between the impaired hand of the participants with stroke and the corresponding hand in NIC subjects (two unpaired tests for LHS and RHS), and between the unimpaired hand of the participants with stroke and the corresponding hand in NIC subjects (two unpaired tests for LHS and RHS). For participants with stroke, the average of both test and retest outcomes was used. Two-sample *t*-tests or Wilcoxon rank sum tests, respectively paired-sample *t*-tests or paired Wilcoxon signed rank tests, were conducted depending on whether data (or their differences for paired testing) were normally distributed or not. Normality was tested with the Shapiro–Wilk or the Shapiro–Francia test, depending on the kurtosis. To correct for multiple comparisons, a Šidák-correction was used. To compare the effect of a LHS versus a RHS on proprioceptive function of the impaired hand (as well as unimpaired hand), their differences to NIC baseline of the corresponding hand were compared in an unpaired test. As NIC baseline the median was used, as the distributions of the left and right DL in NIC subjects were not both normally distributed. Again, the test-retest average was used for the participants with stroke.

Subjects in this study were not age-matched on an individual basis but on a group level by including subjects within the same age range. In return, the sample size of NIC subjects was chosen to be larger, which should improve the estimated distribution of DLs for the same age range. To model neurologically intact performance in elderly, log–normal (semi-infinite positive support) probability density functions were fitted on the DL data of NIC subjects for the right (dominant) and left (non-dominant) hand separately. The 95th percentile was used to characterize impairment in participants with stroke. The CI for the 95th percentile cutoff were calculated by bootstrapping.

The trial duration of the robotic assessment was compared between impaired and unimpaired hands of participants with stroke (averaged test-retest, paired-sample *t*-test or paired Wilcoxon signed rank test, depending on normality of the paired differences), and between NIC subjects (mean of both hands for each subject) and the impaired, respectively unimpaired hand of participants with stroke (averaged test-retest, two-sample *t*-test or Wilcoxon rank sum test, depending on normality of the two distributions). The Šidák-correction method was used to correct for multiple comparisons.

Spearman’s rank-order correlations were calculated for the impaired hand between the DL (test-retest average) and the outcome measures of the clinical scales (i.e., up-down, localization, Von Frey hair, vibration, stereognosis, working memory). As the up-down test also measures proprioception, the correlation with the robotic assessment could be regarded as a test for concurrent validity. The clinical scores for the impaired hand and the working memory test were also compared between the participants with RHS and LHS with two-sample *t*-tests and Wilcoxon rank sum tests, respectively.

The influence of sex on the proprioceptive outcome measures was tested by separately comparing the robotic outcome measures for the dominant as well as the non-dominant hand in male versus female NIC subjects, and for the impaired as well as the unimpaired hand in participants with stroke (two-sample *t*-test or Wilcoxon rank sum test, depending on normality of the distributions).

Significance levels were set to *α*=0.05. Probability values *p*<0.05 and *p*<0.01 are marked as * and **. Descriptive statistics are reported as mean ± SD, unless otherwise stated. For non-parametric statistics the median and interquartile range (IQR) was reported. All statistical analyses were performed in MATLAB R2014a (MathWorks, Natick, MA, USA).

## Results

Twenty-one participants with stroke completed the two sessions of robotic assessments, and two participants with stroke dropped out of the study (P1 was not able to perceive any movements applied by the robotic device, and P6 prematurely quit the study). As reported by the therapist conducting the robotic assessments, one of the 21 participants with stroke (P13) was not able to correctly follow the task instructions and showed severe concentration problems. Therefore, this participant was excluded from all statistical analyses. Of the 20 participants with stroke (65.9±8.3 years, range: [55, 79] years, 12 male, 8 female, pre-stroke handedness: 18 right handed, 2 ambidextrous), 10 suffered from a LHS and 10 from a RHS. Participants with stroke were 43.7±118.8 weeks post lesion (range: [4, 517] weeks). For one chronic participant with stroke (P17) only the year but not the exact lesion date was known. Average number of days between the test and retest of the robotic assessment in participants with stroke was 2±1 days (range: [1, 4] days), and between the clinical assessment and the first robotic assessment was 4±4 days (range: [0, 13] days). The demographics of all the 23 recruited participants with stroke can be found in Table 1. One of the 29 NIC subjects was excluded according to Tukey’s rule. The average age of the remaining 28 NIC subjects (13 male, 15 female, all right handed) was 63.2±6.6 years (range: [55, 80] years).

**Table 1:** Demographics of the participants with stroke

Participant	Age	Gender	Handedness	Lesion	Post lesion	Stroke type and location
with stroke	[years]		(pre-stroke)	side	[weeks]	
P1 (D)	74	M	R	LHS	11	Infarction of the left MCA
P2	56	M	R	RHS	6	ICH in the right frontotemporal region
P3	68	M	R	LHS	5	Hemorrhage in the left basal ganglia
P4	60	F	A	RHS	14	Partial infarction of the right MCA
P5	79	M	R	LHS	4	Hemorrhage in the left basal ganglia with intraventricular extension
P6 (D)	67	M	R	RHS	12	Infarction of the right MCA, with emphasis on the dorsal and cranial
						aspects and involvement of the basal ganglia
P7	57	F	A	LHS	20	Left ACA SAH and cerebral vasospasms with partial infarction in the
						left MCA- and ACA-territory
P8	67	M	R	RHS	6	Infarction of the right MCA
P9	55	M	R	LHS	8	Hemorrhage in left basal ganglia
P10	57	F	R	LHS	14	Left pontine infarction
P11	70	F	R	RHS	6	Right cerebellar infarction
P12	79	M	R	RHS	7	Infarction of the right MCA
P13 (E)	55	M	R	RHS	14	Partial infarction of the right MCA
P14	79	F	R	RHS	517	Hemorrhage in the right basal ganglia
P15	75	F	R	LHS	144	Infarction of the left MCA
P16	62	M	R	RHS	7	Infarction in the right medulla oblongata
P17	72	M	R	LHS	197–249	Left ICH
P18	67	M	R	LHS	6	Hemorrhage in the left basal ganglia
P19	57	F	R	RHS	10	Mixed SAH and ICH of the right ACA
P20	73	M	R	RHS	15	Multiple ischemia in the right MCA-territory
P21	58	F	R	LHS	5	Multiple ischemia in the left MCA- and PCA-territory
P22	67	M	R	LHS	14	Cerebellar (both sides) and left pontine nucleus infarctions
P23	60	M	R	RHS	23	Ischemic infarction in the right vertebrobasilar territory

One participant with stroke (P13) was excluded due to inability to concentrate and follow the task instructions correctly. For participant P17 only the lesion year was known. *Abbreviations*: *D* dropout, *E* excluded, *M* male, *F* female, *R* right handed, *A* ambidextrous, *RHS* right hemisphere stroke, *LHS* left hemisphere stroke, *ACA* anterior cerebral artery, *ICH* intracerebral hemorrhage, *MCA* middle cerebral artery, *PCA* posterior cerebral artery, *SAH* subarachnoid hemorrhage

**Fig. 2:**
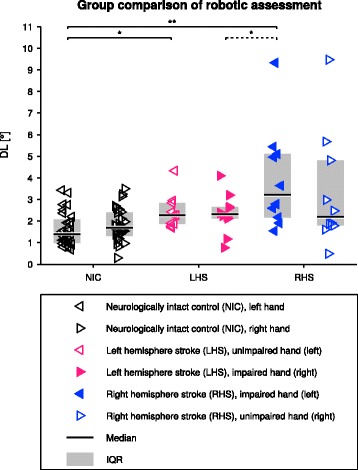
Comparison of the difference limen (DL) of both hands in neurologically intact control (NIC) subjects, participants with left hemisphere stroke (LHS) and right hemisphere stroke (RHS). For the patients, test and retest were averaged for a better DL estimate. The dashed bracket indicates that the statistical test was conducted on baseline-removed data (i.e., using the median for the corresponding hand of the NIC)

The outcomes of the robotic assessment for all groups (NIC, LHS, and RHS) are illustrated for both hands in Fig. 2. Performance of NIC subjects in the robotic proprioception assessment averaged at 1.82° ± 0.77° (median: 1.70°, IQR: [1.31°, 2.40°]) for the right (dominant) hand, and 1.62° ± 0.78° (median: 1.39°, IQR: [0.98°, 2.08°]) for the left (non-dominant) hand. There was no statistically significant difference between the two hands (*t*(27)=1.046,*p*=0.838). No significant effect of sex on the DL was found in NIC subjects (all *p*-values >0.4). Participants with LHS averaged at 2.35° ± 0.94° (median: 2.31°, IQR: [2.12°, 2.65°]) for the impaired (right) hand, and 2.43° ± 0.80° (median: 2.27°, IQR: [1.87°, 2.84°]) for the unimpaired (left) hand. Participants with RHS averaged at 3.95° ± 2.36° (median: 3.21°, IQR: [2.17°, 5.10°]) for the impaired (left) hand, and 3.31° ± 2.66° (median: 2.19°, IQR: [1.79°, 4.81°]) for the unimpaired (right) hand. No significant effect of sex on the DL was found in participants with stroke (all *p*-values >0.2). For participants with LHS, there was no significant difference between their impaired (right) hand and the right hand of NIC subjects (*t*(36)=1.747,*p*=0.373). However, there was a significant difference between their unimpaired (left) hand and the left hand of NIC subjects (*Z*=2.7,*p*=0.038). For participants with RHS, proprioception of the impaired (left) hand was significantly worse than the left hand of NIC subjects (*t*(36)=4.656,*p*<0.001), whereas the unimpaired (right) hand was not significantly different (*Z*=1.8,*p*=0.307). The difference from baseline for the impaired hand in participants with RHS was significantly larger compared to participants with LHS (*t*(18)=−2.384,*p*=0.028). There was no significant difference between the unimpaired hand in participants with RHS compared to participants with LHS (*Z*=−0.9,*p*=0.385). The two log–normal models for neurologically intact proprioception were DL ∼Lognormal(*μ*,*σ*^2^) with *μ*= 0.493 and *σ*= 0.508 for the right, dominant hand and *μ*= 0.381 and *σ*= 0.455 for the left, non-dominant hand. The 95th percentile of NIC subjects was 3.78° (CI: [3.03°, 5.32°]) for the right and 3.10° (CI: [2.47°, 4.01°]) for the left hand. The DL of the left hand was higher than the 95th percentile for 2 NIC subjects. The test-retest average outcome value of the unimpaired (left) hand of participants with LHS was above the impairment cutoff value for 1 participant. Based on a single assessment, 2 (test), respectively 1 (retest), additional participants with stroke would have been considered as impaired. In 5 participants with RHS both test and retest assessments of the impaired (left) hand were above the cutoff value. For the DL of the right hand, this was the case for 1 participant with LHS (impaired hand). The average DLs of the unimpaired (right) hand were above the cutoff in 3 participants with RHS. In one of these participants only the result of one assessment (test) would be considered as impaired. Based on a single assessment, 1 (retest) additional participant with stroke would have been considered as impaired. In 2 participants with RHS both impaired and unimpaired hands were above the 95th percentile.

The values of test-retest reliability for the impaired and unimpaired hand were 0.73 and 0.16, respectively. The detailed descriptive statistics for the DL of the test and retest, reliability, *SEM* characterizing the measurement variability, and *SRD* for evaluating changes are summarized in Table 2.

**Table 2:** Reliability analysis

	DL Test		DL Retest				
	Mean ± SD	Range	Mean ± SD	Range	*ICC*(2,1) [CI]	*SEM*	*SRD*
Impaired	3.08° ± 1.93°	[0.12°, 8.70°]	3.21° ± 2.22°	[0.73°, 9.97°]	0.73 [0.44,0.89]	1.07°	2.95°
Unimpaired	2.90° ± 1.94°	[0.33°, 9.53°]	2.84° ± 3.11°	[0.65°, 15.11°]	0.16 [0.00,0.56]	2.33°	6.45°

Summary of the reliability analysis for the outcome measure of the robotic assessment (i.e., difference limen (DL)) of the impaired and unimpaired hand, respectively. Reported are descriptive statistics for the DL for both test and retest, reliability (*ICC*(2,1) and its 95% CI), standard error of measurement (*SEM*), and smallest real difference (*SRD*)

**Fig. 3:**
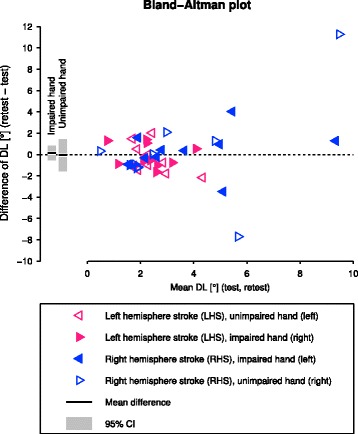
Bland-Altman plot of the test-retest of the robotic assessment in participants with stroke. The bars indicate the mean difference $\bar {d}$ between the difference limen (DL) of the two test sessions (solid black line) and its 95% CI (gray bar) for both impaired and unimpaired hand separately

There were no systematic biases between test and retest, as can be seen by $\bar {d}$ and its 95% CI in the Bland-Altman plot (Fig. 3).

The average number of trials, convergence rate of the PEST algorithm, duration of the assessment, and duration per trial are reported in Table 3. There was no statistically significant difference for the duration per trial between assessments of the impaired and unimpaired hand in participants with stroke (*Z*=1.6,*p*=0.272), nor between assessments in NIC subjects and the impaired (*Z*=0.4,*p*=0.963) or unimpaired (*Z*=1.4,*p*=0.416) hand of participants with stroke, respectively. The inattention detection rate (percentage of cases) based on the psychophysical data and resulting number of excluded trials (around one third of the trials in participants with stroke and one sixth in NIC subjects) are also summarized in Table 3.

**Table 3:** Task execution characteristics and inattention

	Max trials	Trials	Converged	Duration	Duration/trial	Inattention	Trials excluded
Impaired	60	55±5	35%	12.3±1.7 min	13.4±1.6 s	10%	20±8
Unimpaired	60	53±7	45%	11.3±1.8 min	12.8±0.8 s	18%	16±9
NIC, both hands	120	65±18	91%	14.1±3.8 min	13.1±0.7 s	13% (7%)	14±9 (8±2)

Summary of the properties of the PEST sequences, as well as percentage of cases where sustained inattention (distraction from the task) was detected (according to [78]) and resulting number of excluded trials, for participants with stroke (impaired and unimpaired hand) and neurologically intact controls (NIC). For trial- and duration-related results, test and retest were averaged for each participant with stroke, whereas in the case of NIC subjects their left and right hand were averaged. For the results regarding inattention, test and retest were pooled for the participants with stroke, and left and right hand were pooled for the NIC subjects. The values in parentheses correspond to the percentage of cases where sustained inattention was detected and resulting number of excluded trials after truncating the sequences to a maximum of 60 trials

All Spearman’s rank-order correlations between the robotic outcome measure and the clinical scales were weak to fair and not statistically significant (see Table 4). Many clinical assessments showed either floor or ceiling effects, with up to 90% of the data. In particular, in the up-down test, which is the most relevant of the clinical scales as it measures proprioception, all except one participant with stroke reached the highest possible score showing no proprioceptive deficits. Statistical tests did not show any significant differences between participants with RHS and LHS for the clinical assessments (neither for tests assessing the impaired hand (*p*>0.325) nor for the working memory test (*p*=0.121)).

**Table 4:** Clinical assessments and correlations

	Up-down	Localization	Von Frey hair	Vibration	Stereognosis	Working memory
Mean ± SD	9.80±0.63	9.30±1.89	3.72±1.79	7.00±0.94	8.60±0.97	5.15±1.92
Range	[8.00, 10.00]	[4.00, 10.00]	[1.41, 8.27]	[5.00, 8.00]	[8.00, 10.00]	[3.00, 8.00]
Floor/ceiling	0%/90%	0%/80%	0%/0%	0%/30%	0%/30%	0%/0%
Spearman *r*_*s*_(8)	−0.41	0.34	0.16	−0.09	0.34	−0.09
Spearman *p*	0.24	0.34	0.65	0.81	0.33	0.81

Descriptive statistics of the clinical assessments and Spearman’s rank-order correlation (*r*_*s*_, *n*=10) with the average of the difference limen (DL) of test and retest provided by the robotic assessment. Reported correlations are for the impaired (contralesional) side

## Discussion

The aim of this study was to evaluate the psychometric and clinimetric properties (reliability, validity, and clinical feasibility and usability) of a robot-assisted assessment of MCP proprioception, using a 2AFC approach and the adaptive sampling procedure PEST providing the angular joint position DL, in a stroke population. The study demonstrated a good reliability for the contralesional hand in participants with stroke, a difference in proprioceptive function between participants with stroke and NIC subjects, and that participants with RHS may be more affected compared to participants with LHS. Only weak correlations between the robotic outcome measure and clinical scales were obtained, among others due to ceiling effects of the latter. The assessment duration of around 12 min, demonstrated high feasibility of the assessment in an impaired population.

### Test-retest reliability

According to general reliability recommendations (excellent: >0.75, fair to good: 0.4–0.75, poor: <0.4) [87], the intraclass correlation analysis revealed a good reliability for the impaired hand in participants with stroke and a poor reliability for the unimpaired hand. The poor reliability for the unimpaired limb may result from the distribution of data, with LHS and RHS having more similar means and LHS and RHS combined having a smaller IQR compared to the impaired side, as inter-subject variability has an influence on reliability [88]. Therefore, we can recommend to use the proposed assessment as a reliable tool for the impaired, contralesional hand of stroke patients.

Studies evaluating other assessment approaches using various matching paradigms for different upper limb joints [14, 31, 32, 34, 35, 38, 41, 50, 54, 56, 60] or similar joint position DL estimation methods [34, 59] reported coefficients of reliability ranging from fair to excellent for most outcome measures. However, it is difficult to compare results, as other approaches may measure different aspects of proprioception, or because some studies investigated the reliability in NIC subjects instead of in the target population, which may lead to non-representative results, due to different inter- and intra-subject variabilities [88]. Several studies also used inappropriate time intervals between the test and retest (e.g., right after each other without a time interval long enough to prevent recall bias) or suboptimal methods for calculating reliability. In comparison to robotic approaches, the kinaesthetic subscales of the Nottingham Sensory Assessment (finger finding, positional mimicry, and up-down test) have a poor inter-rater agreement (Cohen’s *κ* from 0.26 to 0.39) for the hand [22, 26]. This may originate among others from the nature of the manually applied stimuli which are not well-controlled (e.g., amplitude of movement) and thus may vary across raters.

The Bland-Altman plot showed that there was no systematic bias from test to retest. However, it revealed two outlier data points for the unimpaired hand according Tukey’s outlier test (difference of test-retest >4.08° or <−4.41°). Visual inspection of the PEST sequences showed that for both outliers in one of their two test-retest sequences there was a period of divergence from the threshold, possibly due to inattention (i.e., distraction from the task), which failed to be detected by the inattention detection algorithm [78]. Removing these two outliers from the reliability analysis improved the reliability for the assessment of the unimpaired hand and resulted in a fair reliability (*ICC*(2,1) [CI]: *r*=0.41 [0.00,0.73]).

It should be noted that the *ICC*(2,1) is very sensitive and directly depends on the intra- and inter-subject variability. As a matter of fact, removing participants with stroke P8 with an average DL of 9.33° (test: 8.70°, retest: 9.97°) for the impaired hand, would reduce the test-retest reliability (*ICC*(2,1) [CI]: *r*=0.49 [0.04,0.77]). This shows that reporting the CI for the reliability is also important. Indeed, the reliability without P8 still remains within the CI reported in Table 2. By including also sub-acute participants with stroke in a future cross-sectional study with a larger sample size, we would expect more participants with stroke with high DLs, confirming a good test-retest reliability.

### Construct validity and proprioceptive consequences of stroke

Hypothesis-based comparisons between NIC subjects and participants with stroke, as well as between participants with RHS and LHS could give some indication of validity of the assessment (i.e., construct validity). As a matter of fact, the robotic assessments of proprioception of the present study revealed worse proprioception (around 2.3 times larger DL) in the impaired (left) hand of the RHS group compared to NIC subjects. This is comparable to the results showing a fingertip position DL (flexion–extension) three times larger in participants with stroke compared to the control group when using a Same-Different paradigm [89]. The existence of post-stroke proprioceptive deficits could also be established with various matching studies [14, 31, 47, 90] and studies using other types of proprioceptive measures [54]. While one would also expect the impaired (right) hand of participants with LHS to have a significantly larger DL, this was not the case in our study. When modeling NIC performance, half of the 10 participants with RHS were above the 95th percentile of NIC performance, compared to one out of 10 participants with LHS. This can be due to various reasons. Since this was an exploratory study including participants with stroke suffering from different levels of impairment, some of the included participants with stroke may not have presented important proprioceptive deficits, resulting in a reduced difference between the NIC and stroke groups. The high rate of ceiling effects in the clinical scales (e.g., in the up-down test) would support this hypothesis. Other reasons could be the inclusion of participants with stoke within a broad range of time after stroke, as proprioceptive impairments after stroke can reduce with time [91], lack of sub-acute participants with stroke, and the exclusion of participants with stroke who were not able to detect any manually applied large passive finger movements, which reduces the reported prevalence of proprioceptive deficits. Another hypothesis for this RHS/LHS difference could be that in the right handed participants with RHS, the non-dominant (left) hand is impaired which could potentially suffer more from non-use, compared to the dominant (right) hand which is affected in right handed participants with LHS.

Another reason for more severe proprioceptive deficits in participants with RHS could be the higher ratio of participants with RHS with a cortical stroke when compared to participants with LHS. This could potentially explain a minor neglect or somatosensory deficit in participants with RHS. It is possible that with the screening procedure for neglect some participants with stroke with minor or declining neglect might have also been included in the study. Furthermore, cortical LHS may lead more often to aphasia [92], which could also explain the smaller ratio of participants with LHS with a cortical stroke, as aphasia is an exclusion criterion. When inspecting the stroke type and location, it can be noticed that participants who suffered from ischemia or infarction of the MCA (middle cerebral artery) or in the MCA-territory tend to have among the highest DLs for the impaired hand. All participants with RHS among these fall beyond the 95th percentile of healthy performance. This could be explained by the fact that the majority of the somatosensory cortex is supplied by the MCA [93]. Participants with only partial infarctions of the MCA or in the MCA-territory did not present such high DLs.

While there are several studies with NIC subjects [43, 48, 64, 66, 67] suggesting a left arm/right hemisphere advantage for the processing of proprioceptive inputs in right handed subjects and vice versa for left handed subjects [48, 67], no statistical significant dominance in proprioceptive performance could be shown for the non-dominant hand in NIC subjects of the present study. However, as the values for the mean as well as the median DL were lower for the non-dominant limb, the results tend to support this evidence. This proprioceptive dominance of the non-dominant limb may be attributed to an imbalance of body side representations in the different hemispheres, which, in case of a stroke, could lead to an asymmetric incidence of proprioceptive deficits [94] and more severe proprioceptive deficits when the non-dominant limb is affected. For this reason, when comparing between the performance of the impaired limbs of participants with LHS and RHS in this study, the trend for better proprioception of the non-dominant limb was accounted for by removing the baseline (i.e., the median for the corresponding hand of the NIC). Indeed, our study revealed a significant difference between the impaired limbs of participants with LHS and RHS (all right handed with two exceptions being ambidextrous), the latter showing more severe proprioceptive deficits in accordance to our hypothesis. These findings are in line with other studies showing similar tendencies in different proprioceptive outcome measures in stroke patients [14, 47, 63], and thus may endorse the validity of this assessment.

Our study also showed that the unimpaired hand can be affected after stroke: The group analysis revealed a significant difference for the unimpaired (left) hand of participants with LHS, and three of the participants with RHS had DLs larger than the 95th percentiles of the corresponding hand in NIC subjects. The first could be explained by the fact that LHS subjects had similar medians for both hands, but the NIC subjects showed a trend towards better proprioception in the left hand. In contrast to the impaired hand, no significant difference was found between participants with LHS and RHS for the unimpaired hand. However, there were more participants with RHS with DLs above the 95th percentile compared to participants with LHS. This is in line with the aforementioned hemispheric dominance for proprioception and with existing evidence revealing deficits in the “unimpaired” ipsilesional limb after unilateral cortical lesions [95–97]. Thus, it would be more correct to refer to this limb as “less affected” instead of “unimpaired”. As a consequence, proprioceptive performance of the impaired hand and its recovery progress throughout therapy should be compared to the NIC population instead of to the ipsilesional limb of the participant with stroke.

### Concurrent validity

From all correlations with clinical scales, the best correlation was found between the proprioceptive DL provided by the robotic assessment and the clinical outcome measure of the proprioceptive up-down test. However, since a ceiling effect occurred on the up-down test for all but one participants with stroke (9/10 participants with stroke scored the maximum points), the correlation for this clinical scale should not be over-interpreted. Correlations with all other clinical assessments (not directly targeting proprioception) were also weak to fair. Thus, no concurrent validity could be established by correlating the robotic outcome measure with the clinical scales. This clearly demonstrates the limitations of clinical assessments, unable to sensitively quantify and differentiate proprioceptive deficits, and points out the need for finer-graded proprioceptive assessments as gold standards [30]—a need also identified by therapists [29]. As a consequence of these results, it is not surprising that when comparing the clinical scores between participants with RHS and LHS, no statistically significant differences could be found.

Similar as for the construct validity, the concurrent validity analysis would benefit from the inclusion of sub-acute participants with stroke with more severe proprioceptive deficits. In a future study, different sensitive outcome measures of robotic proprioception assessments using different paradigms but assessing the same proprioceptive aspects could be correlated to circumvent the common problem of coarse clinical scales and further investigate concurrent validity.

### Clinical feasibility and confounds: duration, number of trials, inattention, and memory

The similarity of unconstrained trial duration (i.e., without time limit) between participants with stroke and NIC subjects proves the feasibility of the assessment concept and response interface in participants with stroke (independently of whether the contralesional or ipsilesional hand was tested). The convergence of PEST (around trial 65 in NIC subjects) shows that a maximum number of 60 trials and other termination criteria for PEST are adequately chosen for a clinical application in patients. Since the DL estimate is based on the fit of the psychometric function using data from the full sequence, it is not imperative to obtain convergence of the PEST algorithm to compute the outcome measure. Around 60 PEST trials requiring in total less than 15 min is a good compromise between precision and accuracy of the outcome measures and clinical feasibility, as assessments should be quick to administer [29, 98].

While there exist some position matching assessments which are reported to be more rapid (e.g., [14, 50]), the duration of the assessment should be put into relation with the information content of the outcome measures and the reliability of the assessment. Nevertheless, the present assessment is relatively quick to administer, comparable to recently developed perturbation detection assessments taking 10–15 min [55, 56] and much faster than other assessments using a 2AFC paradigm requiring around 45 min [59].

The results with NIC subjects in Table 3 show that periods of inattention to the task are more frequent in a longer assessment, resulting in the potential collection of biased data and unreliable estimates of proprioception. However, despite the reduction of the assessment time, NIC subjects and participants with stroke still showed periods of inattention. Moreover, the prevalence of inattention was higher in participants with stroke, which is in line with the literature [76, 77]. The inattention confound could be addressed successfully by using the inattention detection algorithm [78]. This is also reflected in the reliability: When not using the algorithm to reduce the introduced bias from inattention to the task, the reliability decreases (*ICC*(2,1) [CI]: *r*=0.63 [0.28,0.84], for the impaired hand). Furthermore, differences between NIC subjects and participants with stroke (partially due to the inattention confound) would increase and could lead to misleading interpretation. Thus, the results presented here (i.e., when using the inattention detection algorithm) are more conservative and should more accurately represent differences in proprioception.

The lack of correlation between the robotic outcome measures and the working memory test, as well as the Bland-Altman analysis and a previously conducted mixed-effects-model on 30 elderly NIC subjects [99] demonstrate the robustness of the assessment against memory, learning, and fatigue confounds, as there were no systematic biases originating from multiple measurements, number of trials, or order of hands assessed.

### Robot-assisted assessment paradigm

Using the 2AFC paradigm with passively presented proprioceptive stimuli makes the proposed method a purely proprioceptive assessment independent of motor function, in contrast to most ipsi- or contralateral matching assessments. Furthermore, it relies neither on interhemispheric transfer for the comparison of stimuli nor on the sensorimotor function of the ipsilesional “unimpaired” limb, which is an advantage, as the central integration of proprioceptive information across the limbs and the ipsilesional limb may also be affected by a cerebral lesion [96, 100], as also found in the present study. This paradigm for the assessment of single-joint proprioception also allows the use of more cost-effective and simpler robotic devices with only one actuated degree-of-freedom, in contrast to sophisticated robotic multi-limb devices or actuated planar end-effector platforms (e.g., [101, 102]) used for other types of proprioceptive assessments [14, 54]. Furthermore, there exists some evidence that after stroke there is a high agreement of somatosensory deficits in neighboring body parts [103], which could render the assessment of multiple joints redundant in many cases.

Compared to other psychophysical paradigms (e.g., Yes-No or Same-Different), 2AFC is more robust against decision criteria (i.e., response bias) [61, 72], is suggested to help directing the subject’s attention to the task [95], and allows measurements of sensitivity to smaller thresholds [61]. In addition, PEST can adapt and converge over a wide range of angle differences without prior knowledge about the subject’s DL, while converging rapidly towards the DL. This leads to shorter assessments with data points covering the regions of interest of the psychometric function.

Presenting passive movements with different amplitudes but same trajectory duration leads to varying peak velocity. Therefore, the subject may partly not only rely on the perception of angular joint position but also movement velocity. This is preferable to a time confound, as primary endings of muscle spindles respond both to the length change and rate of length change of the muscle [104]. As a consequence, the proposed robotic assessment remains a purely proprioceptive test and assesses both subparts of proprioception: limb position sense (sense of stationary position) and kinaesthesia (sense of limb movement) [23]. Other solutions such as using movement velocities below detection threshold [37] would not be feasible in a clinical setting as they would result in an increased assessment duration.

### Limitations of the study

One limitation of the present study is the lack of a sensitive clinical scale as a reference for proprioceptive function. Therefore, some participants with stroke did not present detectable proprioceptive deficits according to the up-down test (as suggested by the high ceiling rate). On the other hand, participants with stroke with the inability to detect any large passive movement were excluded, as they would not be able to perform the robotic assessment. Both of these factors limit the severity range of deficits. As a consequence, the stroke group was more similar to the control group. These points negatively affected the concurrent validity analysis and hypothesis testing (e.g., when comparing participants with stroke to NIC subjects).

Age-matching is important, as proprioception declines with age [33, 35, 37, 40, 42, 48, 52, 53, 99, 105, 106]. By individually age-matching subjects instead of age-matching on a group level, the statistical power could be increased by using paired tests comparing participants with stroke and NIC subjects. On the other hand, age-matching on a group level allows increasing the sample size of the NIC group when creating normative models. The width of the CIs of the 95th percentiles of healthy performance show, however, that a future study would benefit from a larger sample size for better estimates of cutoff values. Thus, the number of participants with stroke suffering from deficits based on the present cutoff value are only indicative and should be interpreted with care.

The reliability analysis would also benefit from a larger sample size [107]. Nevertheless, this exploratory study already demonstrated good reliability when testing the impaired (contralesional) hand of participants with stroke. Based on this promising finding, a new cross-sectional study with a larger sample size can be conducted, including also left handed subjects.

## Conclusion

The evaluation of psychometric and clinimetric properties in this exploratory study including participants with stroke and a group of neurologically intact subjects demonstrated good reliability, validity, and high feasibility of the proposed robot-assisted adaptive assessment of finger proprioception. While existing clinical scales that do not require any tools may be appropriate for some fast preliminary screening of neurological patients, this robotic assessment has the potential to sensitively and accurately quantify proprioceptive deficits. Together with its good usability and short administration time, which facilitate its integration into clinical routine, it becomes a powerful tool for more standardized assessments, for understanding the role of proprioceptive deficits in the functional recovery process, as well as for improving diagnostics, prognostics, monitoring, and planning of sensorimotor rehabilitation programs in patients after neurological injuries.

## Abbreviations

2AFC: Two-alternative forced choice
A: Ambidextrous
ACA: Anterior cerebral artery
CI: Confidence interval
D: Dropout
DL: Difference limen
E: Excluded
F: Female
ICC: Intraclass correlation coefficient
ICH: Intracerebral hemorrhage
IQR: Interquartile range
LHS: Left hemisphere stroke
M: Male
MCA: Middle cerebral artery
MCP: Metacarpophalangeal
NIC: Neurologically intact controls
PCA: Posterior cerebral artery
PEST: Parameter Estimation by Sequential Testing
R: Right handed
RHS: Right hemisphere stroke
SAH: Subarachnoid hemorrhage

## References

[1] Hasan Z (1992). Role of proprioceptors in neural control. Curr Opin Neurobiol.

[2] Sober SJ, Sabes PN (2003). Multisensory integration during motor planning. J Neurosci.

[3] Butler AJ, Fink GR, Dohle C, Wunderlich G, Tellmann L, Seitz RJ, Zilles K, Freund H-J (2004). Neural mechanisms underlying reaching for remembered targets cued kinesthetically or visually in left or right hemispace. Hum Brain Mapp.

[4] Konczak J, Corcos DM, Horak F, Poizner H, Shapiro M, Tuite P, Volkmann J, Maschke M (2009). Proprioception and motor control in Parkinson’s disease. J Mot Behav.

[5] Jeannerod M, Michel F, Prablanc C (1984). The control of hand movements in a case of hemianaesthesia following a parietal lesion. Brain.

[6] Ghez C, Gordon J, Ghilardi MF, Christakos CN, Cooper SE (1990). Roles of proprioceptive input in the programming of arm trajectories. Cold Spring Harb Symp Quant Biol.

[7] Gentilucci M, Toni I, Chieffi S, Pavesi G (1994). The role of proprioception in the control of prehension movements: a kinematic study in a peripherally deafferented patient and in normal subjects. Exp Brain Res.

[8] Carey LM. Somatosensory Loss after Stroke. Critical Reviews™; in Physical and Rehabilitation Medicine. 1995; 7(1):51–91.

[9] Sarlegna FR, Sainburg RL (2009). The roles of vision and proprioception in the planning of reaching movements. Adv Exp Med Biol.

[10] Reding MJ, Potes E (1988). Rehabilitation outcome following initial unilateral hemispheric stroke. Life table analysis approach. Stroke.

[11] Shah SK (1978). Deficits affecting the function of the paralysed arm following hemiplegia. Aust Occup Ther J.

[12] Sullivan JE, Hedman LD (2008). Sensory dysfunction following stroke: Incidence, significance, examination, and intervention. Top Stroke Rehabil.

[13] Schabrun SM, Hillier S (2009). Evidence for the retraining of sensation after stroke: a systematic review. Clin Rehabil.

[14] Dukelow SP, Herter TM, Moore KD, Demers MJ, Glasgow JI, Bagg SD, Norman KE, Scott SH (2010). Quantitative assessment of limb position sense following stroke. Neurorehabil Neural Repair.

[15] Kessner SS, Bingel U, Thomalla G (2016). Somatosensory deficits after stroke: a scoping review. Top Stroke Rehabil.

[16] Kusoffsky A, Wadell I, Nilsson BY (1982). The relationship between sensory impairment and motor recovery in patients with hemiplegia. Scand J Rehab Med.

[17] Feys H, De Weerdt W, Nuyens G, van de Winckel A, Selz B, Kiekens C (2000). Predicting motor recovery of the upper limb after stroke rehabilitation: value of a clinical examination. Physiother Res Int.

[18] Han L, Law-Gibson D, Reding M (2002). Key neurological impairments influence function-related group outcomes after stroke. Stroke.

[19] Abela E, Missimer J, Wiest R, Federspiel A, Hess C, Sturzenegger M, Weder B (2012). Lesions to primary sensory and posterior parietal cortices impair recovery from hand paresis after stroke. PloS ONE.

[20] Smith DL, Akhtar AJ, Garraway WM (1983). Proprioception and spatial neglect after stroke. Age Ageing.

[21] Prescott RJ, Garraway WM, Akhtar AJ (1982). Predicting functional outcome following acute stroke using a standard clinical examination. Stroke.

[22] Lincoln NB, Crow JL, Jackson JM, Waters GR, Adams SA, Hodgson P (1991). The unreliability of sensory assessments. Clin Rehabil.

[23] Gilman S (2002). Joint position sense and vibration sense: anatomical organisation and assessment. J Neurol Neurosurg Psychiatry.

[24] Beckmann YY, Çiftçi Y, Ertekin C (2013). The detection of sensitivity of proprioception by a new clinical test: the dual joint position test. Clin Neurol Neurosurg.

[25] Hirayama K, Fukutake T, Kawamura M (1999). ’Thumb localizing test’ for detecting a lesion in the posterior column-medial lemniscal system. J Neurol Sci.

[26] Lincoln N, Jackson J, Adams S (1998). Reliability and revision of the Nottingham Sensory Assessment for stroke patients. Physiotherapy.

[27] Winward CE, Halligan PW, Wade DT (1999). Current practice and clinical relevance of somatosensory assessment after stroke. Clin Rehabil.

[28] Hillier S, Immink M, Thewlis D (2015). Assessing Proprioception: A Systematic Review of Possibilities. Neurorehabil Neural Repair.

[29] Pumpa LU, Cahill LS, Carey LM (2015). Somatosensory assessment and treatment after stroke: An evidence-practice gap. Aust Occup Ther J.

[30] Suetterlin KJ, Sayer AA (2014). Proprioception: where are we now? A commentary on clinical assessment, changes across the life course, functional implications and future interventions. Age Ageing.

[31] Carey LM, Oke LE, Matyas TA (1996). Impaired limb position sense after stroke: a quantitative test for clinical use. Arch Phys Med Rehabil.

[32] Wycherley AS, Helliwell PS, Bird HA (2005). A novel device for the measurement of proprioception in the hand. Rheumatol (Oxford).

[33] Schmidt L, Depper L, Kerkhoff G (2013). Effects of age, sex and arm on the precision of arm position sense—left-arm superiority in healthy right-handers. Front Hum Neurosci.

[34] Hoseini N, Sexton BM, Kurtz K, Liu Y, Block HJ (2015). Adaptive Staircase Measurement of Hand Proprioception. PLoS ONE.

[35] Kalisch T, Kattenstroth J-C, Kowalewski R, Tegenthoff M, Dinse HR (2012). Age-related changes in the joint position sense of the human hand. Clin Interv Aging.

[36] Scott SH, Dukelow SP (2011). Potential of robots as next-generation technology for clinical assessment of neurological disorders and upper-limb therapy. J Rehabil Res Dev.

[37] Ferrell WR, Crighton A, Sturrock R. D (1992). Age-dependent changes in position sense in human proximal interphalangeal joints. Neuroreport.

[38] Lönn J, Crenshaw AG, Djupsjöbacka M, Johansson H (2000). Reliability of position sense testing assessed with a fully automated system. Clin Physiol.

[39] Lönn J, Crenshaw AG, Djupsjöbacka M, Pedersen J, Johansson H (2000). Position sense testing: influence of starting position and type of displacement. Arch Phys Med Rehabil.

[40] Adamo DE, Martin BJ, Brown SH (2007). Age-related differences in upper limb proprioceptive acuity. Percept Mot Skills.

[41] Juul-Kristensen B, Lund H, Hansen K, Christensen H, Danneskiold-Samsøe B, Bliddal H (2008). Test-retest reliability of joint position and kinesthetic sense in the elbow of healthy subjects. Physiother Theory Pract.

[42] Adamo DE, Alexander NB, Brown SH (2009). The influence of age and physical activity on upper limb proprioceptive ability. J Aging Phys Act.

[43] Adamo DE, Martin BJ (2009). Position sense asymmetry. Exp Brain Res.

[44] Dukelow SP, Herter TM, Bagg SD, Scott SH (2012). The independence of deficits in position sense and visually guided reaching following stroke. J Neuroeng Rehabil.

[45] Gay A, Harbst K, Kaufman KR, Hansen DK, Laskowski ER, Berger RA (2010). New method of measuring wrist joint position sense avoiding cutaneous and visual inputs. J Neuroeng Rehabil.

[46] Squeri V, Zenzeri J, Morasso P, Basteris A. Integrating proprioceptive assessment with proprioceptive training of stroke patients. In: Rehabilitation Robotics (ICORR), 2011 IEEE International Conference On. Zurich: 2011. p. 1–6. 10.1109/ICORR.2011.5975500. 10.1109/ICORR.2011.5975500 22275696

[47] Semrau JA, Herter TM, Scott SH, Dukelow SP (2013). Robotic identification of kinesthetic deficits after stroke. Stroke.

[48] Herter TM, Scott SH, Dukelow SP (2014). Systematic changes in position sense accompany normal aging across adulthood. J Neuroeng Rehabil.

[49] Nomura Y, Ito T. Posture-Angle Perception and Reproduction Characteristics with Wrist Flexion/Extension Motions. In: International Conference on Advances in Computer-Human Interactions (ACHI), 2014. Barcelona, Spain: 2014. p. 154–159.

[50] Rinderknecht MD, Popp WL, Lambercy O, Gassert R. Reliable and Rapid Robotic Assessment of Wrist Proprioception Using a Gauge Position Matching Paradigm. Front Hum Neurosci. 2016; 10(316). 10.3389/fnhum.2016.00316. 10.3389/fnhum.2016.00316 PMC4925678 27445756

[51] Kokmen E, Bossemeyer Jr R, Williams WJ (1978). Quantitative evaluation of joint motion sensation in an aging population. J Gerontol.

[52] Wright ML, Adamo DE, Brown SH (2011). Age-related declines in the detection of passive wrist movement. Neurosci Lett.

[53] Ingemanson ML, Rowe JB, Chan V, Wolbrecht ET, Cramer SC, Reinkensmeyer DJ (2015). Use of a robotic device to measure age-related decline in finger proprioception. Exp Brain Res.

[54] Simo L, Botzer L, Ghez C, Scheidt RA (2014). A robotic test of proprioception within the hemiparetic arm post-stroke. J Neuroeng Rehabil.

[55] Bourke TC, Coderre AM, Bagg SD, Dukelow SP, Norman KE, Scott SH (2015). Impaired corrective responses to postural perturbations of the arm in individuals with subacute stroke. J Neuroeng Rehabil.

[56] Mrotek LA, Bengtson M, Stoeckmann T, Botzer L, Ghez CP, McGuire J, Scheidt RA (2017). The Arm Movement Detection (AMD) test: a fast robotic test of proprioceptive acuity in the arm. J NeuroEngineering Rehabil.

[57] Lambercy O, Juárez Robles A, Kim Y, Gassert R. Design of a robotic device for assessment and rehabilitation of hand sensory function. In: Rehabilitation Robotics (ICORR), 2011 IEEE International Conference on. Zurich: 2011. p. 1–6. 10.1109/ICORR.2011.5975436. http://dx.doi.org/10.1109/ICORR.2011.5975436. 10.1109/ICORR.2011.5975436 22275636

[58] Rinderknecht MD, Popp WL, Lambercy O, Gassert R, Auvray M, Duriez C (2014). Experimental Validation of a Rapid, Adaptive Robotic Assessment of the MCP Joint Angle Difference Threshold. Haptics: Neuroscience, Devices, Modeling, and Applications. Lecture Notes in Computer Science.

[59] Cappello L, Elangovan N, Contu S, Khosravani S, Konczak J, Masia L (2015). Robot-aided assessment of wrist proprioception. Front Hum Neurosci.

[60] Semrau JA, Herter TM, Scott SH, Dukelow SP (2017). Inter-rater reliability of kinesthetic measurements with the KINARM robotic exoskeleton. J NeuroEngineering Rehabil.

[61] Macmillan NA, Douglas Creelman C (2005). Detection Theory: A User’s Guide.

[62] Taylor MM, Douglas Creelman C (1967). PEST: Efficient estimates on probability functions. The Journal of the Acoustical Society of America.

[63] Sterzi R, Bottini G, Celani MG, Righetti E, Lamassa M, Ricci S, Vallar G (1993). Hemianopia, hemianaesthesia, and hemiplegia after right and left hemisphere damage. A hemispheric difference. J Neurol, Neurosurg Psychiatry.

[64] Goble DJ, Lewis CA, Brown SH (2006). Upper limb asymmetries in the utilization of proprioceptive feedback. Exp Brain Res.

[65] Goble DJ, Brown SH (2008). The biological and behavioral basis of upper limb asymmetries in sensorimotor performance. Neurosci Biobehav Rev.

[66] Goble DJ, Brown SH (2008). Upper limb asymmetries in the matching of proprioceptive versus visual targets. J Neurophysiol.

[67] Goble DJ, Noble BC, Brown SH (2009). Proprioceptive target matching asymmetries in left-handed individuals. Exp Brain Res.

[68] Bohannon RW, Smith MB (1987). Interrater reliability of a modified Ashworth scale of muscle spasticity. Phys Ther.

[69] Nasreddine ZS, Phillips NA, Bédirian V, Charbonneau S, Whitehead V, Collin I, Cummings JL, Chertkow H (2005). The Montreal Cognitive Assessment, MoCA: A Brief Screening Tool For Mild Cognitive Impairment. J Am Geriatr Soc.

[70] Gauthier L, Dehaut F, Joanette Y (1989). The Bells Test: A quantitative and qualitative test for visual neglect. International Journal of Clinical Neuropsychology.

[71] Oldfield RC (1971). The assessment and analysis of handedness: the Edinburgh inventory. Neuropsychologia.

[72] Gescheider G (1985). Psychophysics: method, theory, and applications.

[73] Hogan N (1984). Adaptive control of mechanical impedance by coactivation of antagonist muscles. Autom Control, IEEE Trans.

[74] Wald A (1947). Sequential Analysis.

[75] Hall JL (1981). Hybrid adaptive procedure for estimation of psychometric functions. J Acoust Soc Am.

[76] Tuhrim S, Gordon WA (1993). Medical therapy of ischemic stroke. Advances in Stroke Rehabilitation.

[77] Rinne P, Hassan M, Goniotakis D, Chohan K, Sharma P, Langdon D, Soto D, Bentley P (2013). Triple dissociation of attention networks in stroke according to lesion location. Neurology.

[78] Rinderknecht MD, Ranzani R, Popp WL, Lambercy O, Gassert R (2018). Algorithm for improving psychophysical threshold estimates by detecting sustained inattention in experiments using PEST. Attention Perception Psychophysics.

[79] Prins N, Kingdom FAA. Palamedes: Matlab routines for analyzing psychophysical data. 2009. http://www.palamedestoolbox.org. Accessed 7 Jan 2013.

[80] Wichmann FA, Hill NJ (2001). The psychometric function: I, Fitting, sampling, and goodness of fit. Percept Psychophys.

[81] Rolke R, Magerl W, Campbell KA, Schalber C, Caspari S, Birklein F, Treede R-D (2006). Quantitative sensory testing: a comprehensive protocol for clinical trials. Eur J Pain.

[82] Rydel A, Seiffer W (1903). Untersuchungen über das Vibrationsgefühl oder die sog. "Knochensensibilität" (Pallästhesie). Eur Arch Psychiatry Clin Neurosci.

[83] Wechsler D (1997). WAIS-III: Administration and Scoring Manual: Wechsler Adult Intelligence scale.

[84] Shrout PE, Fleiss JL (1979). Intraclass correlations: uses in assessing rater reliability. Psychol Bull.

[85] Lexell JE, Downham DY (2005). How to assess the reliability of measurements in rehabilitation. Am J Phys Med Rehabil.

[86] de Vet HCW, Terwee CB, Knol DL, Bouter LM (2006). When to use agreement versus reliability measures. J Clin Epidemiol.

[87] Fleiss JL (1999). Reliability of Measurement. The Design and Analysis of Clinical Experiments.

[88] Streiner DL, Norman GR (2008). Health Measurement Scales: a Practical Guide to Their Development and Use.

[89] Brewer BR, Klatzky R, Matsuoka Y (2008). Visual feedback distortion in a robotic environment for hand rehabilitation. Brain Res Bull.

[90] Kattenstroth J-C, Kalisch T, Kowalewski R, Tegenthoff M, Dinse HR (2013). Quantitative assessment of joint position sense recovery in subacute stroke patients: a pilot study. J Rehabil Med.

[91] Semrau JA, Herter TM, Scott SH, Dukelow SP (2015). Examining Differences in Patterns of Sensory and Motor Recovery After Stroke With Robotics. Stroke.

[92] Pedersen PM, Stig Jørgensen H, Nakayama H, Raaschou HO, Olsen TS (1995). Aphasia in acute stroke: incidence, determinants, and recovery. Ann Neurol.

[93] Gray H, Standring S, Anand N, Birch R, Collins P, Crossman A, Gleeson M, Jawaheer G, Smith AL, Spratt JD (2016). Gray’s Anatomy: the Anatomical Basis of Clinical Practice.

[94] Vallar G, Antonucci G, Guariglia C, Pizzamiglio L (1993). Deficits of position sense, unilateral neglect and optokinetic stimulation. Neuropsychologia.

[95] Dannenbaum RM, Jones LA (1993). The assessment and treatment of patients who have sensory loss following cortical lesions. J Hand Ther.

[96] Carey LM, Matyas TA (2011). Frequency of discriminative sensory loss in the hand after stroke in a rehabilitation setting. J Rehabil Med.

[97] Jones RD, Donaldson IM, Parkin PJ (1989). Impairment and recovery of ipsilateral sensory-motor function following unilateral cerebral infarction. Brain.

[98] Gresham G, Duncan P, Stason W, Adams H, Adelman A, Alexander D, Bishop D, Diller L, Donaldson N, Granger C, Holland A, Kelly-Hayes M, McDowell F, Myers L, Phipps M, Roth E, Siebens H, Tarvin G, Trombly C (1996). Post-stroke rehabilitation: Assessment, referral, and patient management. Quick Reference Guide for Clinicians, Number 16. J Pharmacoepidemiol.

[99] Rinderknecht MD, Lambercy O, Raible V, Liepert J, Gassert R (2017). Age-based model for metacarpophalangeal joint proprioception in elderly. Clin Interv Aging.

[100] Schaefer SY, Haaland KY, Sainburg RL (2007). Ipsilesional motor deficits following stroke reflect hemispheric specializations for movement control. Brain.

[101] Scott SH (1999). Apparatus for measuring and perturbing shoulder and elbow joint positions and torques during reaching. Journal of Neuroscience Methods.

[102] Scheidt RA, Lillis KP, Emerson SJ (2010). Visual, motor and attentional influences on proprioceptive contributions to perception of hand path rectilinearity during reaching. Exp Brain Res.

[103] Connell LA, Lincoln NB, Radford KA (2008). Somatosensory impairment after stroke: frequency of different deficits and their recovery. Clin Rehabil.

[104] Proske U, Gandevia SC (2012). The proprioceptive senses: their roles in signaling body shape, body position and movement, and muscle force. Physiol Rev.

[105] Stelmach G, Sirica A (1986). Aging and proprioception. AGE.

[106] Fry-Welch D, Campbell J, Foltz B, Macek R (2003). Age-Related Changes in Upper Extremity Kinesthesis. Physical & Occupational Therapy in Geriatrics.

[107] Hopkins WG (2000). Measures of reliability in sports medicine and science. Sports Med.

